# PTEN Inactivation in Mouse Colonic Epithelial Cells Curtails DSS-Induced Colitis and Accelerates Recovery

**DOI:** 10.3390/cancers17142346

**Published:** 2025-07-15

**Authors:** Larissa Kotelevets, Francine Walker, Godefroy Mamadou, Bruno Eto, Thérèse Lehy, Eric Chastre

**Affiliations:** 1 Sorbonne Université, Inserm, Centre de Recherche Saint-Antoine (CRSA), UMR_S938, 75012 Paris, France; 2 Université Paris Cité, Inserm, Centre de Recherche sur l’Inflammation (CRI), UMR_S1149, 75018 Paris, France; francinecombrouze@gmail.com (F.W.); therese.lehy@orange.fr (T.L.); 3 Laboratoires TBC, Laboratory of Pharmacology, Pharmacokinetics, and Clinical Pharmacy, Faculty of Pharmaceutical and Biological Sciences, 59000 Lille, France; godefroymamadou@wanadoo.fr (G.M.); etobr@laboratoires-tbc.com (B.E.)

**Keywords:** PTEN, tumor suppressor, mouse model, colitis, dextran sulfate sodium (DSS), inflammation, wound-healing, junctional complexes, tight junction, intestinal barrier

## Abstract

**Background**: PTEN is a tumor suppressor that controls many pathophysiological pathways, including cell proliferation, differentiation, apoptosis and invasiveness. Although PTEN down-modulation is a critical event in neoplastic progression, it becomes apparent that transient and local inhibition of PTEN activity might be beneficial for the healing process. **Methods**: In the present study, we investigated the impact of PTEN invalidation in mouse intestinal epithelium under a physiological condition and after dextran sulfate sodium (DSS) treatment to induce experimental colitis. PTEN conditional knockout was induced in intestinal epithelial cells after crossing villin-Cre and PTEN^flox/flox^ mice. **Results**: PTEN invalidation alleviates experimental colitis induced by DSS, as evidenced by decreased weight loss during the acute phase, the lower expression of inflammation markers, including the proinflammatory cytokines IFN-γ, CXCL1 and CXCL2, reduced mucosal lesions, and faster recovery after resolution of inflammation. This protective effect might result in part from the sustained proliferation of colonic epithelium, leading to hyperplasia and increased colonic crypt depth under physiological conditions, which was further exacerbated in the vicinity of mucosal injury induced by DSS treatment. Furthermore, PTEN knockout decreased paracellular permeability, thereby enhancing the intestinal barrier function. This process was associated with the reinforcement of claudin-3 immunostaining, especially on the surface epithelium of villin-Cre PTEN^flox/flox^ mice. **Conclusions**: PTEN inactivation exerts a protective effect on the onset of colitis, and the transient and local down-modulation of PTEN might constitute an approach to drive recovery following acute intestinal inflammation.

## Simple Summary

The PTEN tumor suppressor controls a broad range of cellular functions, including cell proliferation, differentiation, migration and apoptosis. The purpose of our study was to delineate the physiopathological role of PTEN in intestinal homeostasis and in susceptibility to colitis. Using a transgenic mouse model, we showed that PTEN invalidation in intestinal epithelium promotes cell proliferation and triggers colonic mucosa hyperplasia. We also evidenced that this invalidation alleviates chemically induced colitis. The underlying mechanisms involved a sustained colonic epithelial cell proliferation in the vicinity of mucosal lesions, favoring re-epithelization, and the enhancement of intestinal barrier function through the strengthening of tight junctions and the reduction of paracellular permeability. Thus, PTEN inactivation exerts a protective effect on the onset of colitis, and the transient and local restraint of PTEN activity might hasten healing following acute inflammation.

## 1. Introduction

Phosphatase and tensin homolog (PTEN) is a tumor suppressor that is inactivated at high frequency in a large number of human sporadic cancers [1,2]. Germline PTEN mutations cause rare autosomal dominant syndromes with a broad clinical phenotypic spectrum, including harmatomas, susceptibility to cancer and autism spectrum disorder [3]. PTEN is a phosphatase that can dephosphorylate not only Ser/Thr and Tyr amino-acid residues in protein substrates, but also the phosphatidylinositol (3,4,5)-trisphosphate produced by phosphatidylinositol 3-kinase (PI3K), hence down-regulating the Akt signaling pathway. PTEN also exerts phosphatase-independent biological activities in the nucleus and in the cytoplasm, such as interacting and stabilizing the tumor suppressor P53, promoting DNA repair, maintaining chromosome integrity and inhibiting cell mobility [4,5,6,7,8]. Thus, PTEN exerts pleiotropic effects on many pathophysiological processes, including cell metabolism, proliferation, differentiation, apoptosis and invasiveness. The activity of this tumor suppressor is, therefore, subjected to fine tuning at different levels, i.e., transcriptional regulation, transcript degradation (miRNA), post-transcriptional modifications (phosphorylation, Ubiquitination, SUMOylation, oxidation), recruitment in macromolecular complexes and subcellular targeting [5,6,9,10,11,12]. In this context, we previously reported that PTEN is anchored to E-cadherin junctional complexes via the MAG1 scaffolding molecule, and that stabilizing adherens junctions through its lipid phosphatase activity inhibits cell invasiveness [13,14,15].

The incidence of PTEN down-modulation in various types of cancer, and the broad spectrum of its activity, highlight the critical role of this tumor suppressor as a powerful gatekeeper of neoplastic progression. Engineered mouse models have proved invaluable in deciphering the signaling pathways controlled by PTEN and the associated phenotypes [16]. In this respect, the use of mouse models with hypomorphic PTEN alleles demonstrated that even a subtle decrease in PTEN expression levels was sufficient to confer cancer susceptibility in a tissue-dependent manner [17]. Thus, many efforts have been devoted to identifying and characterizing PTEN molecular partners and signaling pathways in order to design novel and efficient targeted therapeutic strategies.

On the other hand, it becomes increasingly clear that some disease treatments might benefit from the transient and local inhibition of PTEN activity [18,19]. For instance, VO-OHpic, a PTEN inhibitor, promotes a cardioprotective effect in the murine model of sudden cardiac arrest, as well as in an ex vivo model of myocardial ischemia reperfusion by reducing the apoptosis of cardiomyocytes [20,21]. Similarly, the pharmacological or siRNA down-modulation of PTEN prevents ischemic injury in rats’ brains by activating the AKT/mTOR pathway [22]. In a rat model of brain traumatic injury, treatment with bpV(pic)—another vanadium derivative compound—confers neuroprotection by driving the M2 polarization of microglia to the detriment of the pro-inflammatory M1 phenotype [23]. PTEN inactivation might also rescue defective signaling for angiogenesis, and repair thrombotic microangiopathy [24]. As far as epithelial ulceration is concerned, conditional PTEN knockout in keratinocytes (K14-Cre PTEN^flox/flox^ mice) hastens the healing of skin wounds [25].

Based on these observations, we hypothesized that the cure of inflammatory bowel diseases (IBDs) might also benefit from the control of PTEN activity. This hypothesis was further substantiated by the fact that the oxidative inactivation of PTEN and PTPN12 by NOX1 enhanced wound repair in the DSS experimental mouse model of colitis [26]. IBDs mainly represented by Crohn’s disease and ulcerative colitis are recurrent chronic inflammatory disorders of the human bowel. These pathologies manifest in ulcerative lesions of the mucosa accompanied by a prominent infiltrate of activated cells from both the innate and adaptive immune systems. Disruption of intestinal barrier integrity contributes to the pathogenesis. This barrier involves junctional complexes, including tight junctions and adherens junctions. E-cadherins at adherens junctions ensure the cohesiveness of intestinal epithelial cells, whereas claudins at tight junctions form a dynamic sealing barrier preventing the translocation of microorganisms across the epithelial monolayer. PTEN down-regulation in the human colonic Caco-2 cell line was shown to enhance cell migration, to decrease the expression of claudins 1, 3, 4, 8, and to reduce transepithelial resistance [27]. In contrast, another study using the same cellular model reported that PTEN down-modulation by miR-200c-3p alleviates LPS-induced barrier dysfunction [28].

IBDs are characterized by relapses, followed by periods of remission. Once the inflammation is resolved, mucosal repair occurs through epithelial cell proliferation and migration.

In this context, the present study aimed to investigate the impact of PTEN in intestinal homeostasis and tissue architecture, and its pathophysiological implications in susceptibility to colitis and tissue repair after the resolution of inflammation.

For this purpose, we engineered a mouse model with targeted PTEN invalidation to intestinal epithelial cells, and we investigated the development and the extent of experimental colitis induced by dextran sulfate sodium (DSS).

## 2. Materials and Methods

### 2.1. Animals

All experiments were undertaken in agreement with the European Community Council Directive of 22 September 2010 (010/63/UE) and according to a protocol evaluated and approved by the “Comité d’éthique en matière d’expérimentation animale Paris-Nord C2EA 121”, the Institutional Animal Care and User Ethical Committee of our institution (INSERM and Université Paris Denis Diderot): Approval# 0126.02.

The PTEN^flox/flox^ mice (exons 4 and 5 of PTEN flanked by loxP sites) were generated and kindly provided by Pr Tak W Mak (University of Toronto, Toronto, ON, Canada) [29]. These mice were backcrossed for more than 10 generations with C57Bl6/J (Charles River Laboratories, L’Arbresle, France). Dr Sylvie Robine (Institut Curie, Paris, France) kindly provided us the villin-Cre (B6-Tg(Vil-cre)20Sy/Nci) transgenic mice, previously generated in her Laboratory. These mice express the Cre recombinase selectively in intestinal epithelial cells [30].

The villin-Cre^+/−^ PTEN^flox/flox^ mice were produced by mating PTEN^flox/flox^ animals with villin-Cre^+/−^ mice followed by backcrossing the resulting villin-Cre^+/−^ PTEN^flox/+^ animals with PTEN^flox/flox^. The PTEN^flox/flox^ (villin-Cre^−/−^) littermate were considered as wild-type/control group. Mice were genotyped by PCR amplification of genomic DNA extracted from tail biopsies using the NucleoSpin Tissue kit (Macherey Nagel, Hœrdt, France). For PTEN^flox/flox^, we used the following set of primers: sense 5’ CTC CTC TAC TCC ATT CTT CCC 3’; antisense 5’-ACTCCCACCAATGAACAAAC-3’, generating amplicons of 228 bp and 335 bp for the wild-type and flox/flox alleles, respectively. For villin-Cre, the sense 5’-CAAGCCTGGCTCGACGGCC-3’ and antisense 5’-CGCGAACATCTTCAGGTTCT-3’ primers were used, generating a product of approximately 300 bp.

To minimize cage/microbiota effects, the villin-Cre^+/−^ PTEN^flox/flox^ and control littermates were systematically maintained co-housed. All mice were kept under standard pathogen-free conditions at a temperature of 21–23 °C in ventilated cages with sterile food and water ad libitum, with a 12-h/12-h light/dark cycle. Experiments were performed on animals aged 10–14 weeks.

### 2.2. Colonic Permeability and Transepithelial Electrical Conductance Measurements

Mice were fasted for 16 h with ad libitum access to drinking water. Colons were excised immediately after euthanasia, opened along the mesenteric border and the proximal and distal portions were mounted in 0.16 cm^2^ Ussing chambers (Laboratoires TBC & Biomécatronics SAS, Ruitz, France). Throughout the duration of the experiment, colonic samples were maintained immersed in circulating oxygenated Ringer buffer (pH 7.4) at 37 °C.

For permeability measurements, fluorescein isothiocyanate-dextran 4 kDa for paracellular permeability measurement, or fluorescein isothiocyanate-dextran 70 kDa for transcellular permeability (FD4 and FD70, Sigma Aldrich, Saint-Quentin-Fallavier, France), were added to the mucosal compartment at a final concentration of 250 µM. Four hundred microliters of buffer were collected from the serosal compartment at t0 and after 30, 60 and 90 min with the buffer replacement. Fluorescein fluorescence was measured using a Fluostar plate reader (BMG Labtech, Champignys/Marne, France).

For transepithelial electrical conductance measurements, a short-circuit current (Isc) was applied throughout the experiment via two stainless steel electrodes connected to an automatic voltage-clamp system (JFD–1V, Laboratoires TBC & Biomécatronics SAS, Ruitz, France) to keep the spontaneous transmural electrical potential difference (PD) at 0 mV. Delivered short-circuit current, corrected for fluid resistance, was registered continuously using Biodaqsoft software (V4.2, Laboratoires TBC & Biomécatronics SAS). The Isc (in mA/cm^2^) corresponds to the net ion fluxes transported across the tissue sample in the absence of an electrochemical gradient (mainly Na^+^, Cl^−^ and HCO_3_^−^).

The transepithelial electrical conductance Gt (reverse of resistance), expressed in mS/cm^2^, was calculated according to the equation: Gt = Isc/PD, from the measurements of Isc and PD, recorded at 0, 30, 60 and 90 min [31,32].

### 2.3. Induction of Colitis

Mouse colitis was induced by oral administration for 5 days through a 3% dextran sulfate sodium (DSS, 36–50 kDa; MP Biomedicals, Illkirch, France) solution in sterile drinking water, followed by either 2 (acute phase) or 9 days (recovery phase) of regular sterile drinking water [32]. During the experimental treatments, wild-type and villin-Cre^+/−^ PTEN^flox/flox^ mice were kept co-housed as control or treated groups, and the pathophysiological parameters were blindly evaluated. Animals were monitored daily for weight, water/food consumption and morbidity. The mice were killed at day 7 or at day 14, the colon isolated and, after extensive washing with PBS, opened up, measured and macroscopically examined. Colonic tissue was sectioned along an antero-posterior axis, followed by a cross-section to part the distal and proximal colons. One segment of colonic strips was snap-frozen in liquid nitrogen and cryopreserved for molecular analyses, the 2nd was fixed in formalin (Sigma-Aldrich, St Quentin Fallavier, France) for the histological quantitation of mucosal lesions and mucosal repair, and for immunohistological analysis.

### 2.4. Nucleic Acid Extractions, Reverse Transcription and PCR

Colonic crypts were isolated as previously described [33]. Colons were excised, flushed with physiological saline solution, everted, ligated at one end and filled to distension with a solution containing 2.5 mM EDTA and 0.25 M NaCl (pH 7.5). After 15 min incubation at 4 °C in the EDTA/NaCl solution, colonic crypts were isolated by gentle shaking for 10 s. After each period of shaking, the colon was placed in a fresh medium. Colonic crypts were collected by centrifugation (200× *g*, 2 min), and snap-frozen for subsequent nucleic acid extraction.

Mouse tissues were homogenized using a Polytron homogenizer (Kinematica, Thermo Fisher Scientific, Illkirch, France), and RNA was extracted using NucleoSpin RNA II Kit (Macherey-Nagel). Contaminating eukaryotic DNA was eliminated by DNAse I treatment.

Reverse transcription of total RNA was performed using Moloney murine leukemia virus reverse transcriptase and random primers (Thermo Fisher Scientific, Asnières-sur-Seine, France).

Validation of PTEN recombination in colonic crypts was performed by the multiplex PCR amplification of genomic DNA using the following set of primers: sense 5’-ACAGACCTAGGCTACTGCTC-3’ and antisense 5’-CTAGAAGCAAGACTTCCGTTC-3’ allowing the selective amplification PTEN wild-type allele (amplicon 668bp), and sense 5’-GTCACCAGGATGCTTCTGAC-3’ and antisense 5’-ACTATTGAACAGAATCAACCC-3’ for the recombined PTEN allele depleted of exons 4,5 (849 bp product), respectively. Concurrently, the amplification of the PTEN almost full-length cDNA sequence was performed by RT-PCR, using the following sense 5’-CAAGGATCCACAGCCATCATCAAAGAGATC-3’ and antisense 5’-GTGTTTAAGCTGATCTTCATC-3’, generating products of 1188 bp for wild-type PTEN, and 907 bp for the recombinant sequence.

Real-time PCR was conducted in triplicate using a LightCycler 480 Roche QPCR (Roche Diagnostics, Meylan, France). Primer sequences were based on previous studies, or designed using the Roche assay design center, and synthesis was ordered from Eurogentec (Angers, France). Their sequences are listed in the Supplementary Table S1.

The relative accumulation of the transcripts was determined after the normalization of the threshold cycle (CT) values of the transcripts of interest by subtracting the corresponding CT values of S14, which were selected after comparison of several reference genes and used as the internal standard (ΔCT). The ΔΔCT values were calculated by subtracting the ΔCT values obtained in the transgenic/treated from those obtained in wild-type/untreated mice. The fold changes in mRNA levels were determined using the formula 2^−ΔΔCT^. Statistical analyses were performed on the ΔCT values.

### 2.5. Morphometry, Histological and Immunohistological Analysis

Colonic sections were fixed overnight in formalin, paraffin-embedded and 4-µm sections were stained with either hematoxylin-phloxine-saffron (HPS) or with Periodic acid-Schiff/Alcian blue (PAS + BA) to assess the presence of mucins [32]. Histological longitudinal colonic sections (one section of proximal and distal colon per mouse) were scanned using a VS200 Olympus slide scanner (Evident Scientific France, Rungis, France). The colonic crypts depth was assessed in the distal colon using QuPath software (V0.5.1) [34]. At least 15 well-orientated crypts per section were analyzed.

The histological grading of colitis after DSS treatment was performed blindly through the measurement of epithelial damage, mucosal erosion and ulceration. Data were expressed as a percentage of tissue lesion length in the proximal and distal colons compared to the length of the corresponding tissue sections.

For immunohistochemistry, tissue sections were immunostained with rabbit polyclonal anti-Ki67 antibody (1/50; #RMAB004, Clinisciences, Nanterre, France), anti-claudin-1 antibody (1/100; #RP 153, Diagnostic Biosystems, Diagomics, Blagnac, France), anti-claudin-3 antibody (1/50; #RP 155, Diagnostic Biosystems, France), the mouse monoclonal anti-claudin-2 (1/500; #325600, Invitrogen, Thermo Fisher Scientific, Asnières-sur-Seine, France), anti-β-catenin (1/800; #610154, Transduction Laboratories, BD Biosciences, Le Pont de Claix, France), or anti-E-cadherin (1/100; #610181,Transduction Laboratories, France) using a detection kit (#DS9800, Bond Polymer Refine detection; Leica Microsystems, Nanterre, France). Omission of the primary antibody served as a negative control. To determine the proliferative index, the percentage of Ki67 labeled cells was quantified in at least 15 well-oriented crypts, with lumen apparent from the bottom to the epithelial surface, and with a single layer of cell column.

For the analysis of mucus layers, the distal colon was sliced into rings without preliminary flush, and fixed overnight in Carnoy fixative (60% ethanol, 30% chloroform, 10% acetic acid).

### 2.6. Statistical Analysis

Data set normality was evaluated using Shapiro–Wilk, Anderson Darling, Lilliefors and Jeque-Bera tests, using XLStat software (v2011.4, Addinsoft, Paris, France). Results were expressed as means ± SEM.

Statistical comparisons between control and villin-Cre PTEN^flox/flox^ groups were performed using unpaired two-tailed t tests. Differences between wild-type and villin-Cre PTEN^flox/flox^ mice, treated and untreated, were analyzed by ANOVA, followed by Tukey’s post hoc test, using XLStat software. The level of significance was set at *p* ≤ 0.05.

## 3. Results

### 3.1. Selective PTEN Knockout in Intestinal Epithelial Cells from Villin-Cre PTEN^flox/Flox^ Mice

**Figure 1:**
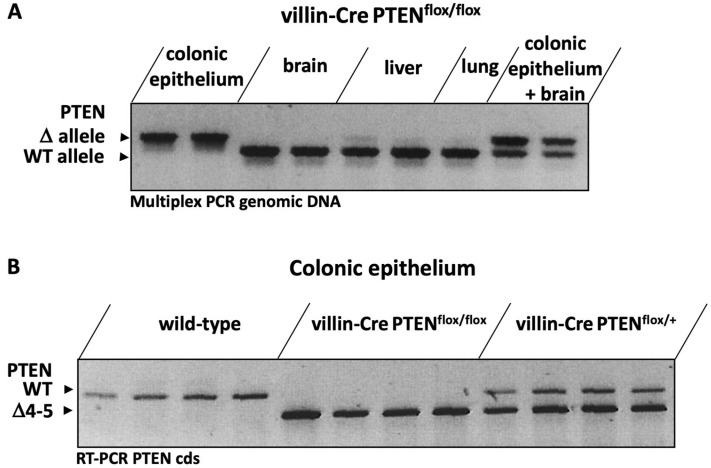
Characterization of the efficiency and the tissue selectivity of PTEN recombination in transgenic mice colonic crypts from villin-Cre PTEN^flox/flox^, as evaluated by PCR. (**A**) Multiplex PCR amplification of the wild-type and recombined PTEN alleles in genomic DNA extracted from colonic epithelium, brain, liver and lung. (**B**) Validation by RT-PCR of PTEN recombination in colonic epithelium from villin-Cre PTEN^flox/+^ and villin-Cre PTEN^flox/flox^ vs. wild type mice. The uncropped photographs of the ethidium bromide-stained agarose gels are shown in Figure S1A,B.

Crossing villin-Cre^+/−^ and PTEN^flox/flox^ mice generated villin-Cre^+/−^ PTEN^flox/+^ animals. This progeny was then backcrossed with PTEN^flox/flox^ to produce villin-Cre^+/−^ PTEN^flox/flox^ (referred to as villin-Cre PTEN^flox/flox^ mice). In a first attempt, we evaluated the efficiency and the tissue selectivity of PTEN recombination par multiplex PCR of genomic DNA. As shown in Figure 1A, the recombined PTEN allele was identified in epithelial cells isolated from colonic mucosa of villin-Cre PTEN^flox/flox^ mice, but was undetectable in brain, lung and liver tissue. Conversely, the wild-type allele was not evidenced in colonic epithelial cells. We further assessed the selectivity of the recombination in colonic crypts isolated from wild-type, heterozygous and homozygous villin-Cre^+/−^ PTEN^flox^ animals by RT-PCR (Figure 1B). As expected, a single RT-PCR product of 1188 bp (corresponding to almost full PTEN cds) was generated from RNA isolated from colonic crypts of wild-type animals, whereas an amplicon of about 900 bp corresponding to the recombinant sequence was identified in colonic crypts from villin-Cre PTEN^flox/flox^ mice. An intermediate pattern was observed with heterozygous animals, where a doublet corresponding to the wild-type and recombined transcripts was evidenced.

### 3.2. Impact of PTEN Depletion on Colonic Architecture and Homeostasis

**Figure 2:**
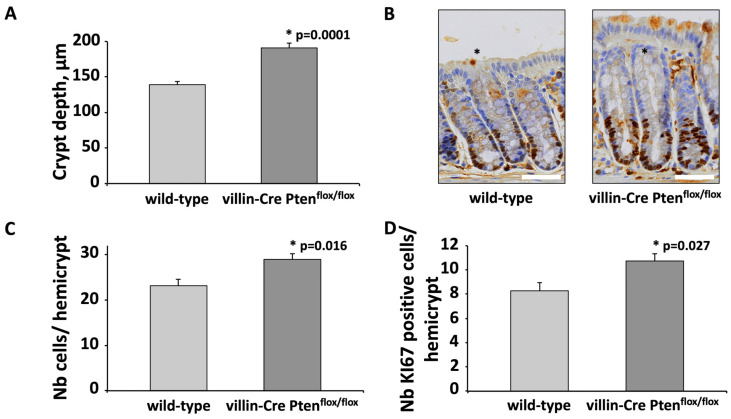
Conditional PTEN invalidation stimulated the proliferation of intestinal epithelial cells and causes colonic hyperplasia. (**A**) Morphometric analysis revealed that PTEN invalidation in colonic epithelial cells increased crypt depth. (**B**) Representative immunostaining of KI67 in the distal colon from villin-Cre PTEN^flox/flox^ mice and control littermate. (*) Well-oriented hemicrypts (**C**) Increased crypt depth in the colonic mucosa of villin-Cre PTEN^flox/flox^ animals results from a greater number of epithelial cells per hemicrypt. (**D**) The number of proliferating colonic epithelial cells (KI67 positive) is higher in PTEN mutant animals. The number of KI67 positive cells and the total number of cells were quantified in at least 15 well-oriented hemicrypts (*) from wild-type (*n* = 5) and villin-Cre PTEN^flox/flox^ (*n* = 6) animals. Statistical differences were determined using two-tailed unpaired *t*-test. Scale bars: 50 µm.

Histological analysis revealed a significant increase in colonic crypt depth of villin-Cre PTEN^flox/flox^ mice as compared to wild-type animals (191 ± 6.5 μm vs. 140 ± 3.7 μm, respectively, *p* = 0.0001) (Figure 2A,B), which was associated with a 26% rise in cell number per hemicrypt, from 23.2 ± 1.5 to 29 ± 1.28 (*p* = 0.016, Figure 2C). To obtain an insight into the underlying mechanism, we investigated cell proliferation using immunostaining of KI67, a nuclear protein expressed in all phases of the cell cycle except G0. As shown in Figure 2B, the proliferative area, which in the intestine is restricted to the base of the crypts, encompassed a significantly higher number of KI67-positive cells per hemicrypt, from 8.3 ± 0.7 in wild-type animal to 10.7 ± 0.61 in villin-Cre PTEN^flox/flox^ mice (*p* = 0.027, Figure 2D).

### 3.3. PTEN Depletion Alleviates Experimental Colitis Induced by DSS

**Figure 3:**
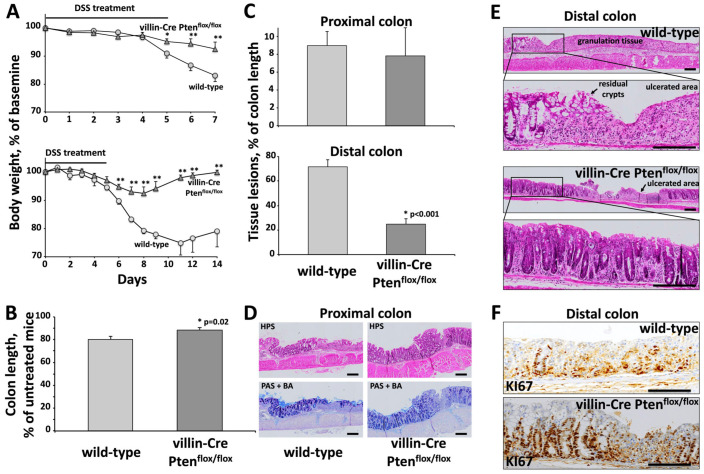
Protective effects of PTEN depletion during the acute and recovery phases of DSS-induced colitis. Pathological features of villin-Cre PTEN^flox/flox^ and wild-type mice. (**A**) Percentage of body weight loss of villin-CrePTEN^flox/flox^ and wild-type mice during the acute phase of colitis (**upper panel**) and during the recovery phase (**lower panel**). Weight loss during DSS administration is a surrogate marker for colitis severity. (**Upper panel**): Values are means ± SEM of one representative experiment out of three. Weight loss was faster in control mice as compared to villin-Cre PTEN^flox/flox^ group (*n* = 11). (**Lower panel**): Percentage of body weight loss of villin-Cre PTEN^flox/flox^ transgenic mice (*n* = 6) and wild-type (*n* = 6) after 5 days of treatment with 3% DSS followed by 9 days of recovery. * *p* < 0.05; ** *p* < 0.01. (**B**) Impact of DSS-induced colitis on colon length in villin-Cre PTEN^flox/flox^ transgenic mice (*n* = 29) and control littermate (*n* = 29) at necropsy on day 7. Colon length relative to untreated animals was significantly shorter in wild-type mice as compared with villin-Cre PTEN^flox/flox^ animals. (**C**–**E**) Histological analysis of tissue lesions as evaluated during acute phase of colitis on day 7. Tissue sections were stained with either HPS, or with PAS + BA, to display neutral and acid mucin expression, respectively. Proximal colon from wild-type (*n* = 21) and villin-Cre PTEN^flox/flox^ (*n* = 16) mice was less affected than the distal part (**upper panel**). Significant erosion of the colonic mucosa was observed in the distal colon of wild-type mice compared with the moderately damaged colons of transgenic mice (*p* < 0.001). (**F**) Immunostaining of KI67 proliferating cells in the colonic mucosa during the acute phase of colitis. The important and sustained proliferation of epithelial cells close to the mucosal erosions in villin-Cre PTEN^flox/flox^ distal colon contrasts with the low KI67 immunoreactivity observed in wild-type mice. Scale bars: 200 µm.

Colitis was induced by 3% DSS administered for 5 days in drinking water, followed by 2 days of recovery. No difference in water consumption was observed in the two groups of animals. As shown in Figure 3A, the development of colitis was associated with progressive weight loss from day 4 to day 7, which corresponds to the acute phase of inflammation. The body weight loss was significantly faster from day 5 in control mice as compared to the villin-Cre PTEN^flox/flox^ group (17 ± 2.17% vs. 7.5 ± 2.49% at day 7, respectively). Furthermore, PTEN depletion resulted in earlier recovery after the acute phase of inflammation (Figure 3A, lower panel).

Further investigations of colonic mucosal injury were performed after necropsy at day 7. Colon shortening is another indicator of disease severity. As shown in Figure 3B, total colon lengths indexed to untreated animals were significantly shorter in control littermate as compared with villin-Cre PTEN^flox/flox^ mice (80.2 ± 2.53% and 88.2 ± 2.27%, respectively, *p* = 0.02). Histological analysis revealed that the proximal colon of both control and PTEN^flox/flox^ animals were slightly affected by DSS treatment (Figure 3C,D). The extent of lesions accounts for 9 ± 1.59% and 7.8 ± 3.14% of proximal colon length, respectively. In contrast, a major erosion of colonic mucosa was observed in the distal colon of wild-type mice (72 ± 5.7% of distal colon length), whereas mucosa of villin-Cre PTEN^flox/flox^ mice was mildly damaged (25 ± 4.4% of distal colon length, *p* < 0.001; Figure 3C,E). Taken together, these data demonstrate the protective effect of PTEN inactivation on DSS-induced colitis.

Reepithelization of superficial wounds and mucosal repair involves the coordinated induction of epithelial cell migration, proliferation and differentiation near the damaged area. The huge and sustained proliferation of epithelial cells adjacent to the mucosal lesions in the villin-Cre PTEN^flox/flox^ distal colon, as evidenced by the strong KI67 immunostaining, likely constitutes a major feature in the mucosal regeneration and faster recovery of mice (Figure 3A,F).

**Figure 4:**
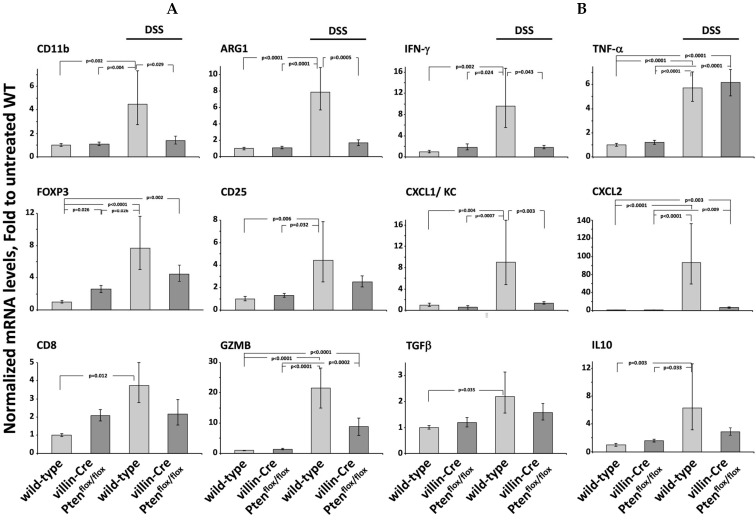
Accumulation of the transcripts encoding cytokines and markers of immune cells in the distal colon of wild-type and villin-Cre PTEN^flox/flox^ mice. (**A**) The accumulation of CD11b, Arg1, FoxP3, CD25, CD8 and GZMB transcripts, expressed by leucocytes, M2 polarized macrophages, T-regulatory/suppressor-T cells, CD8 T cells and NK cells was investigated by qPCR in the distal colon from untreated wild-type and villin-Cre PTEN^flox/flox^ animals, or during the acute phase of DSS-induced colitis. Ct values were normalized with the value measured in the distal colon from untreated wild-type mice, set as reference, and displayed as fold changes (2^−ΔΔCT^). Data are the mean of RT-qPCR performed on RNA samples extracted from 5–6 wild-type and 6 villin-Cre PTEN^flox/flox^ animals. (**B**) Transcript level of the pro-inflammatory IFN-γ, TNF-α, CXCL1/KC and CXCL2/MIP2α, and the anti-inflammatory TGF-β and Il10 cytokines, was quantified as described above. Statistical analysis was performed on the ΔCT values by ANOVA followed by Tukey’s post hoc test.

Since distal colon was mainly injured by DSS-induced colitis, we further compared by qPCR the expression of some cytokines and marker of immune cells in this intestinal segment, under physiological conditions and following challenge with DSS (Figure 4). The pattern of gene expression in the distal colon of DSS-treated wild-type mice revealed a significant increase in the proinflammatory cytokines IFN-γ, CXCL1 and CXCL2, as compared with untreated mice and villin-Cre PTEN^flox/flox^ animals. Similarly, the increase in CD11b, CD8a and granzyme B (GZMB) transcripts evidenced the recruitment of macrophage, neutrophils and cytotoxic T-cells and natural killers (NK). In contrast, high levels of TNF-α were observed in both the control and villin-Cre PTEN^flox/flox^ animals during the acute phase of inflammation.

Under the physiological condition, the slight but significant higher levels of Foxp3 in the distal colon of villin-Cre PTEN^flox/flox^ mice as compared to control animals suggest the presence of immunosuppressive regulatory T cells (T-Reg). This finding is substantiated by a modest but not significant increase in CD25 transcripts, although CD25 expression is not restricted to this subtype of immune cells. Induction of colitis was associated with an increase in FoxP3 and CD25 in the distal colon of control mice, suggesting T-Reg recruitment. Similarly, increased levels of Arginase (Arg1) suggest the presence of M2 macrophages involved in tissue repair. In the same way, high levels of the anti-inflammatory cytokine interleukin 10 (IL10) rise in the colon of control mice during acute inflammation. These results display the marked inflammatory state of the colonic mucosa in wild-type mice, consistent with the extensive mucosal lesions.

### 3.4. Effect of PTEN Depletion on Intestinal Barrier Function

**Figure 5:**
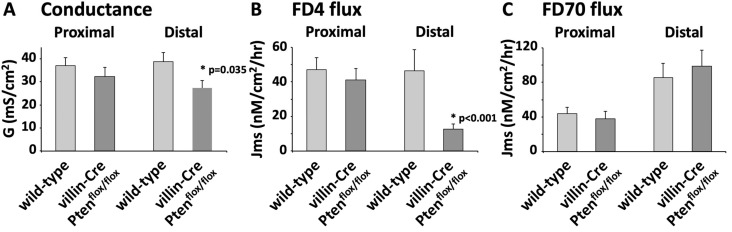
Samples of proximal and distal colon from wild-type and PTEN mutant animals were mounted in Ussing chambers to measure the epithelial conductance (**A**), and the paracellular (**B**) and transcellular (**C**) permeabilities. (**A**) The transepithelial electrical conductance G (reverse of resistance, R) was significantly reduced in the distal colon from villin-Cre PTEN^flox/flox^ mice (*n* = 17), as compared to the control littermate (*n* = 16); (**B**) similarly, the paracellular permeability, as measured by the transmucosal flux (mucosal-to-serosal, Jms) of FD4 flux, was decreased in the distal colon of PTEN mutant mice (wild-type mice, *n* = 9; villin-Cre PTEN^flox/flox^ mice *n* = 8), (**C**), whereas the transcellular flux of FD70 was similar in the two group of animals (*n* = 6, 7). Statistical differences were determined using two-tailed unpaired t-test.

Intestinal barrier function is an interface preventing the translocation of microorganisms and antigens in the mucosa. Impaired intestinal barrier permeability leads to exacerbated sub-epithelial immune system activation and the development of colitis. To determine the effect of PTEN on intestinal barrier permeability, we measured the conductance of the colonic mucosa of wild-type and villin-Cre PTEN^flox/flox^ mice under physiological conditions, using Ussing chambers (Figure 5A). The conductance in the proximal colon was similar in the two groups of animals. In contrast, conductance was significantly lower in the distal colon from villin-Cre PTEN^flox/flox^ as compared to wild-type animals (27.3 ± 3.31 vs. 38.8 ± 4.08 mS/cm^2^, *p* = 0.035). This indicated reduced colonic paracellular permeability, which was further confirmed by the significant decrease in Fluorescein isothiocyanate–dextran (FITC)—labeled dextran 4 kDa (FD4) flux through the distal colon of villin-Cre PTEN^flox/flox^ mice as compared to controls (12.6 ± 2.96 and 46.3 ± 12.45 nM/cm^2^/hr, respectively, *p* < 0.001; Figure 5B). In contrast, the transcellular permeability, quantified using Fluorescein isothiocyanate–dextran 70 kDa (FD70) flux, was unaffected by PTEN depletion (Figure 5C).

**Figure 6:**
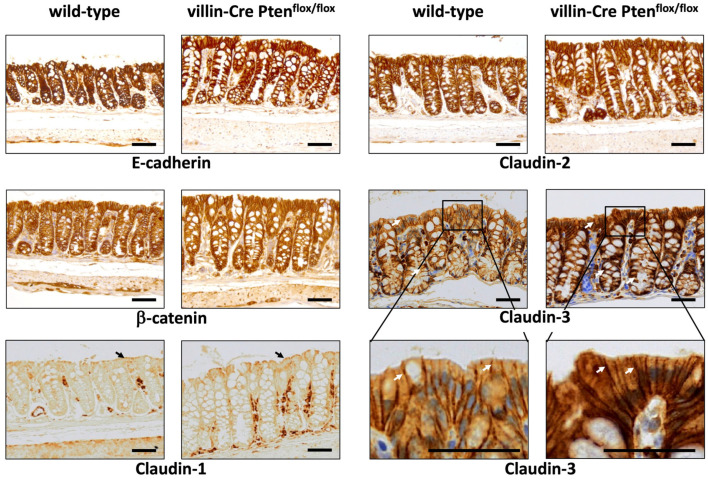
Junctional complex protein immunostaining in the colon of wild-type and PTEN mutant mice. The immunostaining of ß-catenin and E-cadherins, involved in *Adherens junctions*, and of claudin-1, claudin-2 and claudin-3 components of tight junctions was localized at cell-cell contacts, and was similar and uniformly expressed along the crypts for *Adherens junction* proteins and for claudin-2 in the two types of mice on the surface epithelium for claudin-1. A reinforcement of the claudin-3 signal was observed on the surface epithelium of PTEN knockout mice. For each antibody, the immunolabeling was performed simultaneously for all sections using a standard procedure. The arrows display immunolabelling of the surface epithelium for claudin-1, and all along the crypt and on the surface epithelium for claudins-3. Scale bars: 50 µm.

Junctional complexes, mainly tight junctions, control transepithelial permeability. As shown in Figure 6, immunolabelling of E-cadherin and ß-catenin, involved in adherens junction, and of claudin-2—a tight junction-associated protein—were similar in wild-type and villin-Cre PTEN^flox/flox^ animals. Similarly, a faint claudin-1 labelling was observed in both groups of animals, restricted to surface epithelium as previously described [35]. In contrast, claudin-2 and claudin-3 were present along the whole crypt axis and situated at tight junctions and along lateral membranes of epithelial cells. Interestingly, claudin-3 immunostaining was stronger, particularly on the surface epithelium of villin-Cre PTEN^flox/flox^ mice, suggesting that this claudin is involved in the observed decrease in conductance and transepithelial permeability (Figure 6). Accordingly, claudin-3 is considered as a main sealing tight-junction protein, and is highly expressed in the intestine, especially in the distal colon, where the enhanced microorganism concentration requires an efficient barrier function [36,37]. In this connection, the ectopic expression of claudin-3 in the canine kidney epithelial MDCK-II cell line increases transepithelial resistance and reduces FD4 paracellular flux [38]. Furthermore, claudin-3 knockout sensitizes mice to experimental colitis, induced by DSS and by C. rodentium, enhances the proinflammatory cytokine level and promotes dysbiosis [39].

**Figure 7:**
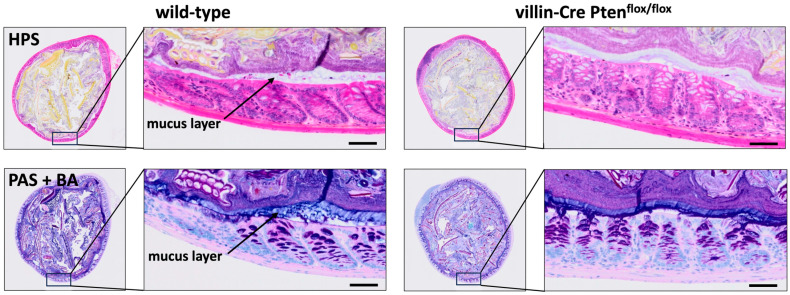
Impact of PTEN depletion on mucus layer thickness. After euthanasia, mouse distal colons were immediately sliced in rings using a scalpel, fixed in Carnoy’s solution, and tissue sections were stained with HPS or PAS + BA to visualize mucus layer. Using this approach, we did not see any change in mucus thickness in wild-type and PTEN mutant animals. Scale bars: 50 µm.

In the distal colon, the mucus layers form a chemical protective barrier towards luminal factors, with the inner layer impenetrable to bacteria. A compromised mucus barrier correlates with the spontaneous development of colitis in the animal model, and disease recurrence for patients with ulcerative colitis [40]. We therefore examined the histological characteristics of colonic mucus in sections of the distal colon fixed with Carnoy’s solution (Figure 7). The examination of these sections from villin-Cre PTEN^flox/flox^ mice and their control littermates stained with HPS or PAS + BA did not reveal any changes in the two groups of animals, suggesting that the differential sensitivity to DSS treatment is not related to a quantitative change in the mucus layer.

## 4. Discussion

In the present study, we demonstrated that targeted PTEN invalidation in mouse intestinal epithelial cells confers resistance to experimental colitis induced by DSS. In addition to the well-documented role of PTEN as a tumor suppressor, our study extends the potential benefit of its down-modulation previously reported for skin, cardiac and brain lesions to intestinal inflammation. To our knowledge, we carried out the first study to selectively assess the role of PTEN in intestinal barrier function and intestinal regeneration in vivo. Our results are consistent with a previous report that indirectly implicated PTEN in mucosal repair after DSS-induced colitis in mice, following its inhibitory oxidation by formyl peptide receptor-induced NOX1 activation and reactive oxygen species production [26]. We evidenced here that this effect relies for a part on an increased epithelial cell proliferation. These observations are in line with the known effects of PTEN in the control of cell growth. The lipid-phosphatase activity of PTEN, and the subsequent down-modulation of the PI3K/AKT signaling pathway, were initially involved in the PTEN-induced G1 growth arrest of glioma cell lines [41]. Later on, additional antiproliferative effects independent of PTEN phosphatase activity were unveiled. These include nuclear PTEN interaction with anaphase-promoting complex/cyclosome (APC/C) E3 ligase, leading to the formation of the APC/C-CDH1 (CDC20-like protein 1) complex and the degradation of polo-like kinase 1 and Aurora kinases [42], as well as p53 interaction in the nucleus [43,44]. It is noteworthy that PTEN plasticity occurs naturally to adapt the cell response to physiological conditions. This includes reversible PTEN inactivation following oxidation by reactive oxygen species, or other post-translational modifications, e.g., phosphorylation, ubiquitination and SUMOylation that regulate activity, stability and subcellular localisation [9,10,11,12].

The intestinal epithelium is in constant cycles of renewal, cellular proliferation, differentiation and senescence. We showed here that, under physiological conditions, PTEN knockout triggers colonic hyperplasia characterized by increased cellularity and crypt depth. In line with these observations, previous studies have reported an extended proliferative area in small intestinal crypts of PTEN knockout mice, associated with an increased number of intestinal stem cells [45,46]. Despite the enhanced number of cycling cells, we did not observe any change in the proliferative index in villin-Cre PTEN^flox/flox^ mice and their control littermates (around 36% KI67 positive cells). This suggests that the relative rate of migration along the crypts and the fate of colonic cells were not markedly affected by PTEN invalidation. In contrast, under DSS-treatment, epithelial cell proliferation in areas close to injured colonic mucosa was further exacerbated in PTEN mutant animals, hence favoring the re-epithelization of superficial wounds. Accordingly, the underlying mechanisms of mucosal repopulation require the orchestrated motility, proliferation and differentiation of epithelial cells in the vicinity of the damaged area, and the crosstalks with other cellular players, including infiltrating myofibroblasts and immune cells, and the release of growth factors and cytokines [47].

Another interesting finding of our study is that, under physiological conditions, PTEN depletion is associated with an increased transepithelial resistance of the colonic mucosa. Intestinal epithelial cells constitute the physical barrier between the lumen and the underlying mucosal immune systems [37]. The maintenance of its integrity is of prime importance to prevent the translocation of microorganisms and their toxins, and to impair dysregulated immune response. Junctional complexes, including adherens juctions and tight junctions, achieve intestinal epithelial cell cohesion. The increased resistance evidenced here results from lower paracellular permeability, as demonstrated by the decreased transmucosal flux of dextran-4000. Tight-junctions are essential for controlling paracellular permeability. These structures involve the clustering and interaction of the transmembrane proteins, claudins, from neighboring cells that are anchored to the actin cytoskeleton through scaffolding molecules, such as ZO1 or MAGI1. Claudins located at the basolateral membrane might also be involved in intestinal homeostasis [48]. Among claudins, claudin-3 is considered as a sealing claudin, as opposed to the channel-forming claudin-2, and can form homophylic and heterophylic interactions. A decreased accumulation of both claudin-3 transcripts and proteins has been evidenced in colonic samples from patients with IBD and from animal models of colitis [35,36,39]. We showed here that claudin-3 immunostaining was reinforced, especially in the surface epithelium of PTEN knockout mice. Interestingly, plasma levels of PTEN and claudin-3 were inversely correlated in the plasma from patients with prostate cancer [49]. Our results are consistent with the increased transepithelial resistance and reduced permeability to FD4 of MDCK-II cells transfected with a claudin-3 expression vector [38]. In the same the way, claudin-3 knockout mice showed an enhanced susceptibility to DSS-induced colitis and to dysbiosis [39].

Our data contrast with the decreased expression of claudins 1, 3, 4 and 8, and the enhanced migration and invasiveness of the Caco-2/15, HCT116 and CT26 colon cancer cell lines following shRNA-induced PTEN down-regulation [27]. Theses discrepancies might be related to the genetic and cellular contexts, i.e., normal vs. cancer cells, the respective activities of signaling pathways and the level and pattern of expression of cell junction proteins. In this context, it should be mentioned that, according to the cell model, claudin-3 expression triggers opposite phenotypes. Epidermal growth factor (EGF) promotes the accumulation of claudin-3 in the human colon cancer HT-29 cell line [50]. In this model, claudin-3 overexpression promotes anchorage-dependent and -independent colony formation, whereas, silencing curtails EGF-induced cell growth. On the other hand, Lin et al. reported that the down-modulation of claudin-3 and claudin-4 in ovarian cell lines triggers Epithelial-Mesenchymal Transition through the activation of the PI3K/AKT pathway [51]. Likewise, claudin-3 knockout in mice induced the Wnt/β-catenin signaling pathway, which was further exacerbated by impaired intestinal barrier function and inflammation, hence promoting colon cancer development [52]. Such divergences are also apparent for adherens junctions. We and others have demonstrated the critical role of the lipid phosphatase activity of PTEN in strengthening E-cadherin junctional complexes [13,14,15,53]. In contrast, PTEN overexpression in endometrial carcinoma cell lines promotes epithelial mesenchymal transition through the β-catenin/Slug-mediated suppression of E-cadherin expression [54]. Furthermore, although PTEN counteracts PI3K activity, PI3K is required for adherens junction formation [55].

Mucus constitutes an additional protective barrier against luminal factors. This is illustrated by the spontaneous development of colitis in mice invalidated for Muc-2, the main mucin expressed in the intestine [56]. This chemical barrier encompasses two layers, the inner layer forms a dense stratified gel that sticks to the surface epithelium, excluding bacteria from the epithelium. The outer layer is much looser, as the result of commensal bacteria colonization and the proteolytic cleavage of mucus gel [37,57]. We did not observe significant changes in the accumulation of Muc-2 transcripts (not shown), nor differences in the thickness of the mucus layer in the distal colon from PTEN mutant mice and control littermates under physiological conditions, suggesting that the mucus barrier was not the main determinant in the differential response between the two groups of animals to DSS-induced colitis. Nevertheless, in the small intestine, where gobblet cells are very sparse, a 1.5-fold increase in their relative number was reported in the jejunum of PTEN mutant mice as compared with control animals (0.12% vs. 0.8% of epithelial cells) [45]. DSS was shown to affect the inner mucus layer, allowing bacterial penetration [58]. An interesting feature that was not addressed in our study is the role of commensal flora in mice’s response to experimental colitis. A series of studies performed in Dr Rhee’s Lab have demonstrated that, on the one hand, PTEN invalidation in intestinal epithelial cells increased mouse sensitivity to Salmonella Typhimurium infection [59], and on the other this invalidation decreases the abundance of Akkermansia muciniphila, a phylia that degrades mucin and might trigger chronic intestinal inflammation [60].

With regard to inflammation, proinflammatory cytokines, such as TNF and IFN-γ, down-regulate tight-junctions and promote intestinal permeability. PTEN is a known inhibitor of the IFN-γ pathway [61]. Nevertheless, since in our model PTEN invalidation is restricted to intestinal epithelial cells, the reduced accumulation of the IFN-γ transcript observed in DSS-treated PTEN mutant mice likely reflects the rapid recovery of the intestinal mucosa. Similarly, CXCL1 and CXCL2 levels in PTEN mutant mice normalized more quickly compared to wild-type animals. In inflamed colonic mucosa, these cytokines are produced by myofibroblasts, epithelial, enteroendocrine and immune cells, including macrophages and granulocytes, and recruit neutrophils whose excessive activation cause tissue injury. PTEN depletion in intestinal epithelial cells might reshape the microenvironment, hence favoring the resolution of inflammation and tissue repair. Accordingly, decreased PTEN expression in ectopic endometrial stromal cells triggers M2 macrophage polarization [62], whereas, PTEN-deficient tumors are characterized by reduced levels of CD8, CD4 and NK cells, and the enhanced infiltration of immunosuppressive cells, i.e., myeloid-derived suppressor cells (MDSCs) and T-regs [63].

Nevertheless, we are aware of several limitations to our study. Firstly, we used one experimental model of colitis in mice. Further studies using other experimental models and/or colonic biopsy explant cultures from IBD patients could strengthen our observations. Secondly, as mentioned above, the microenvironment, including the microbiota, but also myeloid and lymphoid immune cells, is an important element in triggering intestinal inflammation that was not directly addressed in this study. In this respect, PTEN controls both the innate and adaptive immune responses [64]. Thirdly, we need to assess the risk of tumor development associated with long-term PTEN inactivation in intestinal epithelial cells.

## 5. Conclusions

In the present study, we observed that constitutive PTEN invalidation in intestinal epithelium triggers colonic hyperplasia. Nevertheless, PTEN deficiency exerts a protective effect on acute intestinal inflammation and accelerates recovery. Thus, the transient and local down-modulation of PTEN or its downstream effectors appears to be an attractive therapeutic approach to favor healing following acute intestinal inflammation. This could be achieved by using PTEN inhibitors (bpV(phen), BpV(pic)), or by targeting the many effectors systems that control PTEN activity [9,10,12]. Accordingly, PTEN is subjected to a series of post-translational modifications, including phosphorylation, oxidation SUMOYlation and ubiquitination, whose effectors might be selectively chemically addressed. The challenge for such a therapeutic strategy will concern the definition of the length and treatment window, and epithelial cell targeting to avoid any side effect.

## Data Availability

The data are not publicly available due to privacy restrictions.

## Supplementary Materials

The following supporting information can be downloaded at: https://www.mdpi.com/article/10.3390/cancers17142346/s1, Supplementary Table S1: sequences of the primers used in this study for real-time PCR. Figure S1. Original agarose gel Figures.

## Abbreviations

The following abbreviations are used in this manuscript:

BA	Blue alcian
DSS	Dextran sulfate sodium
FD4	Fluorescein isothiocyanate–dextran (FITC)-labeled dextran 4 kDa
FD70	Fluorescein isothiocyanate–dextran (FITC)-labeled dextran 70 kDa
HPS	Hematoxylin-phloxine-saffron
MDSCs	Myeloid-derived suppressor cells
NK	Natural killer
PAS	Periodic acid-Schiff
PI3K	Phosphatidylinositol-4,5-bisphosphate 3-kinase
PTEN	Phosphatase and tensin homolog
T-reg	Regulatory T cells
