# Supplementary material for: PTEN Inactivation in Mouse Colonic Epithelial Cells Curtails DSS-Induced Colitis and Accelerates Recovery
Source: Cancers (Basel). 2025 Jul 15;17(14):2346. doi: 10.3390/cancers17142346 (PMC12293889; doi:10.3390/cancers17142346)
Supplement: Supplementary file 1 [file cancers-17-02346-s001.zip › Table S1.pdf]

Supplementary Table S1. Sequences of the primers used in this study for real-time PCR

| **Gene Name** | **Symbol** | **NCBI Accession nb** | **Sense primer** | **Antisense primer** |
|---|---|---|---|---|
| Arginase 1 | Arg1 | NM007482.3 | 5’- cctgaaggaactgaaaggaaag -3' | 5’- ttggcagatatgcagggagt -3' |
| CD25 antigen, interleukin 2 receptor subunit alpha | CD25/IL2RA | NM_008367.3 | 5'- ccaacacagtctatgcaccaa -3' | 5'- agattctcttggaatcttcatgttc -3' |
| CD8 antigen, alpha chain | Cd8a | NM001081110.2 | 5’- ctcacctgtgcaccctacc -3' | 5’- atccggtccccttcactg -3' |
| Chemokine (C-X-C motif) ligand 1 | Cxcl1 | NM008176.3 | 5’- ggattcacctcaagaacatccagag -3' | 5’- caccсttctactagcacagtggttg -3' |
| Chemokine (C-X-C motif) ligand 2 | Cxcl2 | NM009140.2 | 5’- aggctacaggggctgttgtg -3' | 5’- cgtcacactcaagctctggat -3' |
| Forkhead box P3 | Foxp3 | NM001199347.1 | 5’- tcaggagcccaccagtaca -3' | 5’- tctgaaggcagagtcaggaga -3' |
| Granzyme B | GZMB | NM_013542.3 | 5'- gctgctcactgtgaaggaagt -3' | 5'- tggggaatgcattttaccat -3' |
| Integrin alpha M | Itgam | NM001082960.1 | 5’- caatagccagcctcagtgc -3' | 5’- gagcccaggggagaagtg -3' |
| Interferon gamma | Ifng | NM008337.3 | 5’- cagcaacagcaaggcgaaa -3' | 5’- ctggacctgtgggttgttgac -3' |
| Interleukin 10 | Il10 | NM010548.2 | 5’- cagagccacatgctcctaga -3' | 5’- gtccagctggtcctttgttt -3' |
| Ribosomal protein S14 | Rps14 | NM020600.4 | 5’- caggaccaagacccctgga -3' | 5’- atcttcatcccagagcgagc -3' |
| Transforming growth factor, beta 1 | Tgfb1 | NM011577.1 | 5’- tggagcaacatgtggaactc -3' | 5’- gtcagcagccggttacca -3' |
| Tumor necrosis factor | Tnf | NM013693.3 | 5’- aggctgccccgactacgt -3' | 5’- gactttctcctggtatgagatagcaaa -3' |
