# Supplementary figures and images for: PTEN Inactivation in Mouse Colonic Epithelial Cells Curtails DSS-Induced Colitis and Accelerates Recovery
Source: Cancers (Basel). 2025 Jul 15;17(14):2346. doi: 10.3390/cancers17142346 (PMC12293889; doi:10.3390/cancers17142346)

PCR

Flox / +

Colonic epithelium

Flox / Flox

Colonic  
epithel

Brain

Liver

Lung

Brain +  
Colonic epithel

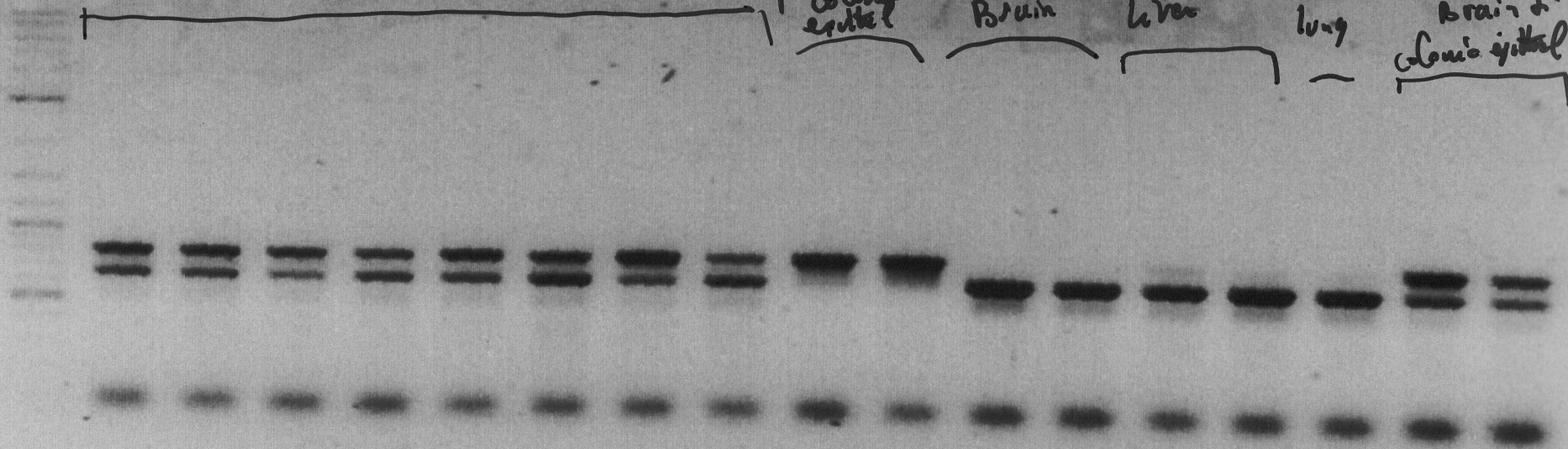

Supplement: Supplementary file 1 [file cancers-17-02346-s001.zip › Figure S1A. PCR Multiplex.pdf]

Colonic  
epithelium

wt

Flx/Flx

Flx/+

wt

Flx/Flx

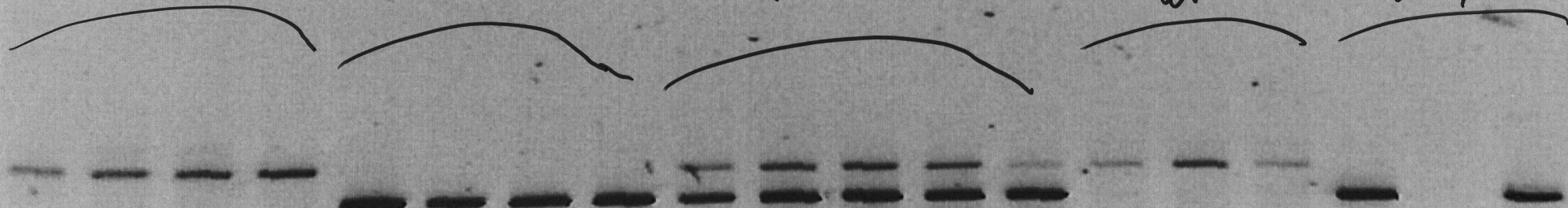

Supplement: Supplementary file 1 [file cancers-17-02346-s001.zip › Figure S1B. RT-Pten.pdf]
